# Elevated Plasma IL-37, IL-18, and IL-18BP Concentrations in Patients with Acute Coronary Syndrome

**DOI:** 10.1155/2014/165742

**Published:** 2014-03-06

**Authors:** Qingwei Ji, Qiutang Zeng, Ying Huang, Ying Shi, Yingzhong Lin, Zhengde Lu, Kai Meng, Bangwei Wu, Kunwu Yu, Meng Chai, Yuyang Liu, Yujie Zhou

**Affiliations:** ^1^Department of Cardiology, Beijing Anzhen Hospital, Capital Medical University, Beijing, China; ^2^Department of Cardiology, The People's Hospital of Guangxi Zhuang Autonomous Region, Nanning, China; ^3^Institute of Cardiovascular Diseases, Union Hospital, Tongji Medical College, Huazhong University of Science and Technology, Wuhan, China; ^4^Department of Ultrasound, The People's Hospital of Guangxi Zhuang Autonomous Region, Nanning, China

## Abstract

*Objective.* More recently, evidence showed that the novel anti-inflammatory cytokine interleukin- (IL-) 37 was expressed in the foam-like cells of atherosclerotic coronary and carotid artery plaques, suggesting that IL-37 is involved in atherosclerosis-related diseases. However, the plasma levels of IL-37 in patients with acute coronary syndrome (ACS, including unstable angina pectoris and acute myocardial infarction) have yet to be investigated. *Methods.* Plasma IL-37, IL-18, and IL-18BP levels were measured in 50 patients with stable angina pectoris (SAP), 75 patients with unstable angina pectoris (UAP), 67 patients with acute myocardial infarction (AMI), and 65 control patients. *Results.* The plasma IL-37, IL-18, and IL-18BP levels were significantly increased in ACS patients compared to SAP and control patients. A correlation analysis showed that the plasma biomarker levels were positively correlated with each other and with the levels of C-reactive protein (CRP), *N*-terminal probrain natriuretic peptide (NT-proBNP), and left ventricular end-diastolic dimension (LVEDD) but negatively correlated with left ventricular ejection fraction (LVEF). Furthermore, the plasma IL-37, IL-18, and IL-18BP had no correlation with the severity of the coronary artery stenosis. *Conclusions.* The results indicate that the plasma IL-37 levels are associated with the onset of ACS.

## 1. Introduction

Acute coronary syndrome (ACS) is the umbrella term for the clinical signs and symptoms of myocardial ischemia including unstable angina pectoris and acute myocardial infarction, which mainly resulted from a disruption of a coronary atherosclerotic plaque associated with partial or complete thrombotic vessel occlusion. It is well accepted that inflammation plays a critical role in the progression of atherosclerosis, plaque instability, and the subsequent onset of ACS.

Interleukin- (IL-) 37, formerly known as IL-1F7, is a novel anti-inflammatory cytokine in the IL-1 ligand family that consists of 11 members and is the only IL-1 family member that is not found in mice [1–3]. Some IL-1 family members, such as IL-1 and IL-18, are well-known proinflammatory cytokines, and they contribute to the atherosclerotic process and onset of ACS [4–7]. Other family members, such as IL-1R*α*, are anti-inflammatory cytokines that efficiently abrogate the pathogenic activity of IL-1 and attenuate the size of atherosclerotic lesions. The role of IL-33, another member of the IL-1 family, seems more complicated. IL-33 positively induces Th2-type responses, and the proinflammatory role of IL-33 has been verified in Th2 cell-mediated inflammatory diseases, such as asthma [8]. In contrast, IL-33 has been verified to inhibit the inflammatory response in atherosclerosis by promoting a Th1-to-Th2 switch and increasing the number of regulatory T (Treg) cells [9, 10]. IL-37 has five splice variants (IL-37a-e) and IL-37b is the main isoform that exists in peripheral blood. IL-37 can decrease the production of proinflammatory cytokines and protect mice from inflammatory and autoimmune diseases [1, 11–13]. More recently, increased il-37 levels have been associated with many chronic inflammatory and autoimmune diseases such as systemic lupus erythematosus and Guillain-barré syndrome in humans [14–16].

In previous studies, we have found that the levels of some anti-inflammatory cytokines related to Treg cells were significantly decreased in ACS patients [17, 18]. Specifically, the levels of plasma IL-35 were positively correlated with the left ventricular ejection fraction, indicating that the plasma IL-35 levels could be a potential biomarker for coronary artery disease (CAD). Recently, unpublished evidence showed that IL-37 was expressed in the foam-like cells of atherosclerotic coronary and carotid artery plaques, suggesting that IL-37 is involved in atherosclerosis-related diseases [2]. Therefore, we hypothesize that as a novel anti-inflammatory cytokine, IL-37 may take part in the onset of ACS, and we measured the levels of plasma IL-37, IL-18, and IL-18 binding protein (IL-18BP) in patients with ACS in the present study.

## 2. Methods

### 2.1. Patients

We recruited 257 patients who underwent diagnostic coronary angiography between October 2011 and October 2012 in the People's Hospital of Guangxi Zhuang Autonomous Region, China. Patients were classified into 4 groups: (1) stable angina pectoris (SAP) (35 men and 15 women, mean age 64.3 ± 12.1), inclusion criteria: typical exertional chest discomfort that was associated with down sloping or horizontal ST-segment depression >1 mm in an exercise test; (2) unstable angina pectoris (UAP) (50 men and 25 women, mean age 64.0 ± 11.2) inclusion criteria: chest pain at rest with definite ischemic electrocardiographic changes: ST-segment changes and/or T-wave inversions; (3) acute myocardial infarction (AMI) (48 men and 19 women, mean age 62.6 ± 10.5), inclusion criteria: myocardial infarction that was confirmed by a significant increase of troponin I and Creatine Kinase MB levels; (4) control group, which consisted of 65 subjects with normal coronary artery (47 men and 18 women, mean age 62.2 ± 8.8).

Written informed consent was obtained from each patient. The study was approved by the Ethics Committee of Beijing Anzhen Hospital, the People's Hospital of Guangxi Zhuang Autonomous Region, and Union Hospital. Patients with valvular heart disease, thromboembolism, collagen disease, disseminated intravascular coagulation, advanced liver disease, renal failure, malignant disease, or septicemia or that were on steroid therapy were excluded from the study.

### 2.2. Blood Samples

In the AMI group, blood samples were obtained from the patients upon arrival into the emergency unit. Fasting blood samples were obtained the morning following admission for the rest of the study groups. The samples were collected into sodium heparin Vacutainers (Becton-Dickinson). The peripheral blood mononuclear cells (PBMCs) were prepared by Ficoll density gradient for analysis by real-time polymerase chain reaction (PCR). Blood was centrifuged for 10 min at 2000 ×g and plasma was stored at −80°C until further use.

### 2.3. ELISA Detection of the Levels of IL-37, IL-18, and IL-18BP

The levels of plasma IL-37 (Adipogen AG, Liestal, Switzerland), IL-18 (MBL, Nagoya, Japan), and IL-18BP (RayBiotech, Norcross GA, USA) were measured by an enzyme-linked immunosorbent assay (ELISA), following the manufacturer's instructions. The minimal detectable concentrations were 10 pg/mL for IL-37, 12.5 pg/mL for IL-18, and 20 pg/mL for IL-18BP. The ELISA intra-assay and interassay coefficients of variation were <5% and <10%, respectively. All of the samples were measured in duplicate.

### 2.4. IL-37, IL-18, and IL-18BP Expression Determined by Real Time-PCR

Total RNA was isolated from freshly PBMCs using a RNeasy kit. cDNA was synthesized using random hexamer primers and RNase H-reverse transcriptase (Invitrogen, USA). Relative quantitative real-time PCR was performed using SYBR-green I Premix ExTaq on the ABI Prism 7900 (Applied Biosystems, Foster, CA) following the manufacturer's instructions. The specific primers were as follows: IL-37: sense: 5′-AACCCCAGTGCTGCTTAGAA-3′, and antisense: 5′-CCCAGAGTCCAGGACCAGTA-3′; IL-18: sense: 5′-TGCATCAACTTTGTGGCAAT-3′, and antisense: 5′-ATAGAGGCCGATTTCCTTGG-3′; IL-18BP: sense: 5′-ACGTCGTCACTCTCCTGGTC-3′, and antisense: 5′-AGCTCAGCGTTCCATTCAGT-3′. The quality of cDNA subjected to the RT-PCR was controlled by amplification of transcripts of GAPDH. GAPDH was analyzed using the following primers: sense: 5′-GAGTCAACGGATTTGGTCGT-3′, and antisense: 5′-GACAAGCTTCCCGTTCTCAG-3′. Quantitative PCR was performed on ABI PRISM 7900 Sequence Detector system (Applied Biosystems) using SYBR Green I Assay (Takara Biotechnology). Relative gene expression level (the amount of target, normalized to endogenous control gene) was calculated using the comparative Ct method formula 2^−ΔΔCt^.

### 2.5. Doppler Echocardiography

Patients underwent M-mode and 2D-echocardiography using a GE ViVid E7 ultrasonography machine (GE Healthcare, America) with a transthoracic 1.5–4.3 MHz probe (M5S-D). Left ventricular end-diastolic diameter (LVEDD) and fractional shortening were measured. Left ventricular ejection fraction (LVEF) was calculated from apical four chambers position by the area-length method.

### 2.6. Gensini Score

The severity of coronary stenosis in patients was estimated with a Gensini coronary score following coronary angiography [19]. The Gensini score was computed by assigning a severity score to each coronary stenosis according to the degree of luminal narrowing and its geographic importance. The reduction in the lumen diameter and the roentgenographic appearance of concentric lesions and eccentric plaques were evaluated (reductions of 25%, 50%, 75%, 90%, and 99% and complete occlusion were assigned Gensini scores of 1, 2, 4, 8, 16, and 32, resp.). The score was then multiplied by a factor that incorporates the importance of the lesion's position in the coronary arterial tree as follows: 5 for the left main coronary artery; 2.5 for the proximal left anterior descending coronary artery (LAD) or left circumflex artery (LCX), 1.5 for the mid-LAD; and 1 for the distal LAD, the right coronary artery, or the mid-distal LCX.

### 2.7. Statistical Analysis

All of the data were given as the mean ± SD. When comparing only 2 groups, Student's *T*-test was used. For comparisons involving 3 or more groups, one-way ANOVA followed by Neuman-Keuls post hoc test was used. Spearman's correlation was used to calculate the correlations between the plasma biomarker levels and the other measured parameters. In all of the tests, a value of *P* < 0.05 was considered to be statistically significant.

## 3. Results

### 3.1. Baseline Characteristics

There was no significant difference in age, gender, history of hypertension, diabetes, or smoking in these four groups. The levels of C-reactive protein (CRP),* N*-terminal probrain natriuretic peptide (NT-proBNP), which is the critical biomarkers for clinical application in CAD [20, 21], and the Gensini score in CAD was significantly higher than that of the control group, whereas the LVEF in CAD was lower than that of the control group. The other parameters including lipid and lipoprotein fractions, fasting glucose, and medications are listed in Table 1.

### 3.2. Plasma Biomarkers in Each Group

**Figure 1:**
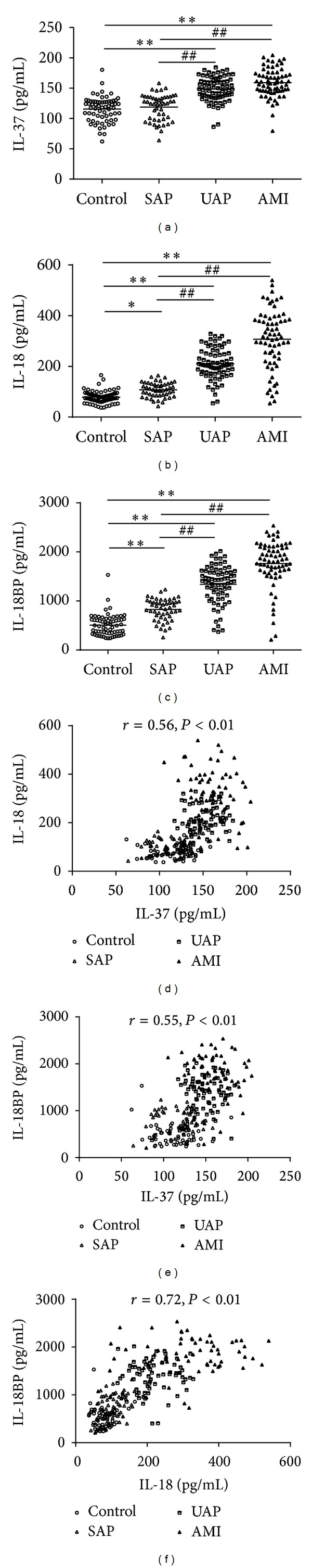
The plasma IL-37, IL-18, and IL-18BP levels in each group. (a) The plasma IL-37 levels in patients with AMI and UAP were significantly increased compared with those of the control and SAP groups. (b) The plasma IL-18 levels in patients with AMI and UAP were significantly increased compared with those of the control and SAP groups, and the plasma IL-18 levels in patients with SAP were significantly increased compared with those of the control group. (c) The plasma IL-18BP levels in patients with AMI and UAP were significantly increased compared with those of the control and SAP groups, and the plasma IL-18BP levels in patients with SAP were significantly increased compared with those of the control group. (d) The levels of IL-37 were positively correlated with the levels of IL-18 (*r* = 0.56, *P* < 0.01). (e) The levels of IL-37 were positively correlated with the levels of IL-18BP (*r* = 0.55, *P* < 0.01). (f) The levels of IL-18 were positively correlated with the levels of IL-18BP (*r* = 0.72, *P* < 0.01). *P < 0.05 versus Control, **P < 0.01 versus Control, ^##^ *P* < 0.01 versus SAP.

As shown in Table 2 and Figure 1, the plasma IL-37, IL-18, and IL-18BP levels in patients with AMI and UAP were significantly increased compared with those of the control group and the SAP group. A correlation analysis showed that IL-37, IL-18, and IL-18BP levels were positively correlated with one another (Figure 1). The plasma IL-37, IL-18, and IL-18BP levels in 192 patients with CAD according to medication have been summarized and the results showed that the administration of aspirin, *β*-blocker, angiotensin-converting enzyme inhibitor (ACEI), angiotensin receptor blocker (ARB), calcium channel blocker (CCB), nitrate and statin had no significant effects on the plasma levels of IL-37, IL-18, and IL-18BP (Table 3). In addition, blood samples were obtained from the culprit coronary artery using manual thrombectomy device (Export AP Aspiration Catheter, Medtronic, USA) and the median cubital vein before the percutaneous coronary intervention procedure in 20 patients with ST-elevation AMI. The results showed that there was no significant difference in plasma IL-37, IL-18, and IL-18BP levels between the culprit coronary artery (162.31 ± 17.34 pg/mL, 296.34 ± 119.93 pg/mL, 1780.64 ± 376.49 pg/mL, resp.) and the median cubital vein (155.81 ± 29.58 pg/mL, 290.52 ± 115.19 pg/mL, 1712.21 ± 433.52 pg/mL, resp.). 257 patients were divided into a hypertensive group (157 cases) and a normotensive group (100 cases). As shown in Table 4, there was no significant difference in plasma IL-37, IL-18, and IL-18BP levels between the hypertensive group and the normotensive group. There was no significant difference in plasma IL-37, IL-18, and IL-18BP levels between the diabetic group (84 cases) and the nondiabetic group (173 cases), and between the smoking group (101 cases) and nonsmoking group (156 cases) (Table 4).

### 3.3. Plasma Biomarkers and Other Measured Parameters

We assessed whether the plasma IL-37, IL-18, and IL-18BP levels were associated with lipid and lipoprotein fractions (triglycerides, high-density lipoprotein cholesterol and low-density lipoprotein cholesterol), fasting glucose, CRP, NT-proBNP, LVEF, LVEDD, and the Gensini score in patients with CAD. The results showed that the levels of IL-37, IL-18, and IL-18BP were positively correlated with CRP, NT-proBNP, and LVEDD but negatively correlated with the LVEF in patients with CAD (Table 5).

### 3.4. Expression of IL-37, IL-18, and IL-18BP in PBMCs

**Figure 2:**
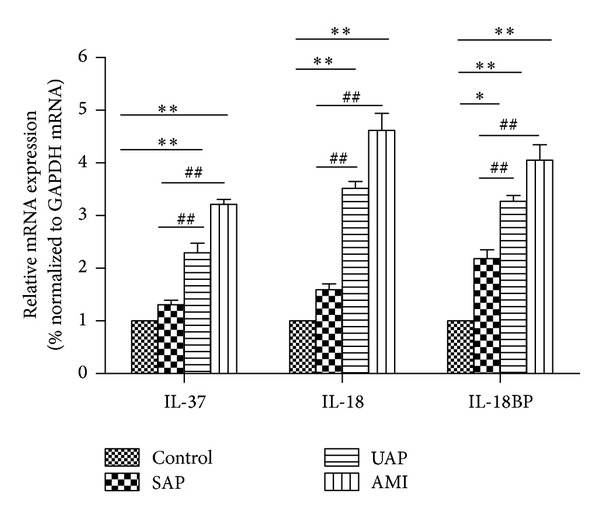
The expression of IL-37, IL-18, and IL-18BP in PBMCs was markedly higher in the AMI and UAP groups than those in the control group, and the expression of IL-18BP in the SAP group was higher than that in the control group. *P < 0.05 versus Control, **P < 0.01 versus Control, ^##^ *P* < 0.01 versus SAP.

As shown in Figure 2, the expression of IL-37, IL-18, and IL-18BP in PBMCs was markedly higher in the AMI group (IL-37: 3.21 ± 0.10; IL-18: 4.62 ± 0.33; IL-18BP: 4.05 ± 0.30) and the UAP group (IL-37: 2.30 ± 0.18, IL-18: 3.52 ± 0.13: IL-18BP: 3.27 ± 0.11) than those in the control group, and the expression of IL-18BP in the SAP group (2.18 ± 0.17) was higher than that in the control group.

## 4. Discussion

In this study, the plasma levels of IL-37, IL-18, and IL-18BP were investigated in ACS patients. Similar to previous studies [7, 22], the levels of plasma IL-18 and IL-18BP were dramatically increased in patients with ACS compared with the control group and the SAP group. In addition, the results showed that the plasma levels of IL-37 and the IL-37 expression were significantly increased in patients with UAP and AMI compared with the control group and the SAP group. A correlation analysis showed that the levels of IL-37 were positively correlated with IL-18, IL-18BP, CRP, NT-proBNP, and LVEDD but negatively correlated with LVEF in CAD patients. Some previous studies showed that the levels of inflammatory cytokine were significantly higher in the culprit coronary artery than those in peripheral blood; some studies showed there was no difference, while some studies found that the levels of inflammatory cytokine were significantly lower in the culprit coronary artery than in peripheral blood [23–26]. In the present study, there was no significant difference in the levels of IL-37, IL-18, and IL-18BP between the culprit coronary artery and the peripheral blood. These results amplified the concept that atherosclerosis is a systemic inflammatory disease rather than a local inflammatory disease. However, the sample size is small and the exact meaning of this phenomenon remains unclear.

IL-37 was identified as a natural suppressor of innate inflammatory responses. IL-37 is expressed in a variety of normal human tissues, such as the lymph nodes, thymus, and uterus [3]. Some IL-37 isoforms are expressed in a tissue-specific fashion. IL-37c is mainly present in the heart, whereas IL-37d and e are only present in the bone marrow and testes [27]. IL-37 is expressed at low levels in peripheral blood mononuclear cells (PBMCs) and dendritic cells (DCs) and is upregulated in an inducible manner. After treatment with phorbol myristoyl acetate (PMA), the mRNA expression of IL-37 was increased 2-fold in PBMCs and 4.5-fold in DCs [3]. In addition, IL-37 is mainly induced in an inflammatory context. IL-1*β*, IL-18, TNF-*α*, IFN-*γ*, and TGF-*β* increase IL-37 synthesis, whereas IL-4 plus granulocyte-macrophage colony-stimulating factor (GM-CSF) inhibit IL-37 expression [1]. It is notable that IL-1*β*, IL-18, TNF-*α*, and IFN-*γ* play critical pathogenic roles in atherosclerosis, and increased levels of these cytokines are associated with the onset of ACS [4–7]. TGF-*β* is an antiatherosclerotic cytokine that stabilizes the lesion but is sharply decreased in ACS, whereas IL-4 has a minor role in the atherosclerotic process [18, 28, 29]. In this study, we found that the levels of IL-37 positively correlated with the increased IL-18 and CRP levels, which are the biomarkers of inflammation, indicating that an increase in IL-37 levels may be resulted from excessive inflammatory response in ACS. Nold et al. found that inhibition of endogenous IL-37 with siRNAs in human PBMCs increased the production of IL-1*β*, IL-6, and TNF, suggesting that IL-37 is crucial for inflammatory control [1]. IL-37 significantly suppresses the production of proinflammatory cytokines and the activation of DCs [1]. The activation of mature DCs is critical for T-cell activation and the production of Th1 and Th17 cytokines, which possess potentially pathogenic properties in atherosclerosis and atherosclerosis related disease [29–32]. In contrast, the results from our group and other groups showed that inhibition of DCs activation significantly suppressed Th1 and Th17 response, induced potent Treg cells, and ameliorated atherosclerosis in atherosclerotic model [33, 34]. Therefore, IL-37 may attenuate atherosclerosis and atherosclerosis related diseases through the inhibition of DCs activation.

A clinical study from Mallat et al. [7] first found that plasma IL-18 levels were significantly increased in CAD, especially in ACS, and significantly correlated with LVEF in CAD patients, which we also observed in this study. IL-18 is produced by monocytes/macrophages, DCs, and several nonhematopoietic cell types. Because IL-18 is an important cytokine that promotes Th1 and natural killer (NK) cell activity, IL-18 is likely to be a key mediator in atherosclerotic plaque instability [35]. In addition, IL-18 increases the expression of certain inflammatory cytokines and MMPs in endothelial cells, SMCs, and monocytes/macrophages, leading to amplification of systemic inflammatory responses. Blocking the effects of IL-18 reduces the atherosclerotic lesion size and induces a switch to a stable plaque phenotype [36, 37]. Evidence shows that IL-18 upregulates the production of proinflammatory cytokines and inducible nitric oxide, which have been associated with the myocardial contractile depression and a loss of cardiomyocytes [7, 38–41]. In addition, IL-18 has the potential to promote cardiomyocyte apoptosis and cardiomyocyte arrhythmogenicity and therefore plays a crucial role in myocardial functional recovery and remodeling [42, 43]. Accumulating evidence suggests that IL-18 levels are not only associated with short-time congestive heart failure (CHF), reinfarction, and the composite end point of cardiovascular death/CHF/MI but also with long-time mortality in ACS patients [44]. Although some studies have found that IL-18 levels were significantly increased in Chinese patients with ACS, evidence that relates IL-18 levels to the prognosis of CAD is still scarce and requires further investigation.

IL-18BP is a soluble, IL-18-binding protein with a high affinity for IL-18 and IL-37. Not high doses but low doses of IL-18BP plus IL-37 effectively reduce the production of inflammatory cytokines induced by IL-18* in vitro*, suggesting a complex role of IL-18BP in inflammatory regulation [45, 46]. Narins et al. found that patients with a recent myocardial infarction had significantly higher IL-18 levels and IL-18/IL-18BP ratio than patients without a recent myocardial infarction but no change in IL-18BP levels [22]. In this study, we found that IL-18BP levels were sharply increased in patients with CAD, especially in those with ACS. This difference could be due to patient selection and the time of blood sample collection. Our sampling time scale was controlled within 24 h after admission, whereas the Narins time scale was delayed 7 to 10 days after symptom onset, indicating that those patients had been treated for a period of time, and therefore the baseline levels of IL-18BP may have been affected. Furthermore, our study revealed that the IL-18BP levels were negatively correlated with LVEF, suggesting a critical role of IL-18BP in myocardial function of CAD.

## 5. Conclusions

In summary, the results of our study first demonstrated that the levels of the novel anti-inflammatory cytokine IL-37 were dramatically increased and negatively correlated with LVEF in ACS patients. However, what role the change in IL-37 levels plays in the development of atherosclerosis and the onset of ACS remains uncertain. In the present study, we also found the levels of IL-18BP, another endogenous antagonist to IL-18 activity, were not only higher in ACS but also negatively correlated with LVEF in CAD patients. However, the present study has some limitations. There has been no follow-up with these ACS patients to assess the short- and long-term prognostic significance of IL-37 and IL-18BP levels, which should be investigated in the future. Since IL-37 is not found in mice, exogenous IL-37 (e.g., recombinant human interleukin-37) and endogenous IL-37 model (e.g., IL-37 transgenic mice) can be used to clarify the role of IL-37 in atherosclerosis.

## Figures and Tables

**Table 1:** Clinical characteristics of patients.

Characteristics	Control	SAP	UAP	AMI
	(*n* = 65)	(*n* = 50)	(*n* = 75)	(*n* = 67)
Age (years)	62.2 ± 8.8	64.3 ± 12.1	64.0 ± 11.2	62.6 ± 10.5
Sex (male/female)	47/18	35/15	50/25	48/19
Hypertension, *n* (%)	41 (63.1)	29 (58)	46 (61.3)	41 (61.2)
Diabetes, *n* (%)	16 (24.6)	14 (28)	29 (38.7)	25 (37.3)
Smoking, *n* (%)	21 (32.3)	20 (40)	36 (48)	24 (35.8)
TC (mmol/L)	4.47 ± 0.93	4.09 ± 0.95	4.37 ± 1.28	4.34 ± 0.87
TG (mmol/L)	1.47 ± 0.72	1.68 ± 0.91	2.00 ± 1.56*	1.58 ± 0.99
LDL-C (mmol/L)	2.75 ± 0.85	2.43 ± 0.88	2.62 ± 1.20	2.69 ± 0.87
HDL-C (mmol/L)	1.32 ± 0.61	1.17 ± 0.42	1.21 ± 0.58	1.02 ± 0.33*
GLU (mmol/L)	5.49 ± 1.50	5.68 ± 2.19	6.60 ± 3.40	6.64 ± 2.76
Creatinine (*μ*mol/L)	76.93 ± 16.68	92.14 ± 28.69*	95.84 ± 34.62*	94.01 ± 38.89*
CRP (mg/L)	1.80 ± 1.22	2.34 ± 1.37	4.67 ± 5.12	6.73 ± 5.23
NT-proBNP (pg/mL)	106.1 ± 44.0	168.4 ± 58.2	365.5 ± 172.2	729.1 ± 326.5
LVEF (%)	65.7 ± 5.0	60.4 ± 8.7*	58.4 ± 10.2*	45.9 ± 10.5*
LVEDD (mm)	46.7 ± 3.2	49.5 ± 6.9	49.8 ± 6.7*	55.1 ± 5.9*
Gensini score	0	30.03 ± 25.14*	32.65 ± 26.05*	69.33 ± 54.91*
Medications, *n* (%)				
Aspirin	12 (18.5)	25 (50)	60 (80)	38 (56.7)
*β*-blocker	15 (23.1)	18 (36)	29 (38.7)	23 (34.3)
ACEI/ARB	32 (49.2)	20 (40)	35 (46.7)	35 (52.2)
CCB	37 (56.9)	25 (50)	48 (64)	18 (26.9)
Nitrate	21 (32.3)	26 (52)	58 (77.3)	31 (46.3)
Statin	19 (29.2)	32 (64)	54 (72)	31 (46.3)

The data are given as the mean ± SD or number of patients. SAP: stable angina pectoris; UAP: unstable angina pectoris; AMI: acute myocardial infarction; TC: total cholesterol; TG: total triglycerides; LDL-C: low-density lipoprotein cholesterol; HDL-C: high-density lipoprotein cholesterol; GLU: fasting glucose; LVEF: left ventricular ejection fraction; ACEI: angiotensin-converting enzyme inhibitor; ARB: angiotensin receptor blocker; CCB: calcium channel blocker.

*P < 0.05 versus control.

**Table 2:** Plasma biomarker levels in each group.

	Control	SAP	UAP	AMI
	(*n* = 65)	(*n* = 50)	(*n* = 75)	(*n* = 67)
IL-37 (pg/mL)	115.47 ± 20.59	118.93 ± 21.46	148.67 ± 19.73^∗∗,#^	159.01 ± 23.23^∗∗,#^
IL-18 (pg/mL)	78.88 ± 25.13	107.82 ± 28.20*	210.60 ± 60.96^∗∗,#^	307.13 ± 117.00^∗∗,#^
IL-18BP (pg/mL)	506.59 ± 217.35	826.68 ± 219.24**	1339.17 ± 381.87^∗∗,#^	1760.14 ± 477.75^∗∗,#^

Note: the data are given as the mean ± SD. *P < 0.05 versus Control, **P < 0.01 versus Control, ^#^ *P* < 0.01 versus SAP.

**Table 3:** Plasma biomarker levels in CAD patients according to medication.

Medication	No.	IL-37 (pg/mL)	IL-18 (pg/mL)	IL-18BP (pg/mL)
Aspirin				
Yes	123	142.66 ± 25.78	219.86 ± 112.11	1341.34 ± 544.37
No	69	147.87 ± 27.86	213.35 ± 110.18	1372.71 ± 499.77
*β*-blocker				
Yes	70	145.36 ± 25.40	221.81 ± 114.09	1398.88 ± 503.72
No	122	144.06 ± 27.34	215.06 ± 109.87	1326.06 ± 541.18
ACEI/ARB				
Yes	90	145.95 ± 26.73	226.23 ± 115.97	1423.76 ± 535.22
No	102	143.29 ± 26.54	209.83 ± 106.75	1289.83 ± 515.40
CCB				
Yes	91	141.77 ± 24.59	203.74 ± 102.97	1310.23 ± 499.44
No	101	147.03 ± 28.17	229.94 ± 117.20	1390.80 ± 551.56
Nitrate				
Yes	115	146.11 ± 27.60	210.40 ± 95.20	1311.60 ± 486.26
No	77	142.18 ± 25.01	228.16 ± 131.44	1413.86 ± 582.02
Statin				
Yes	117	142.72 ± 25.02	215.40 ± 103.75	1334.14 ± 514.75
No	75	147.37 ± 28.82	220.82 ± 122.52	1381.43 ± 549.49

Note: the data are given as the mean ± SD.

**Table 4:** Plasma biomarker levels in traditional risk factors.

	No.	IL-37 (pg/mL)	IL-18 (pg/mL)	IL-18BP (pg/mL)
Hypertension	157	137.72 ± 28.67	181.59 ± 115.14	1149.17 ± 610.33
Normotension	100	136.33 ± 27.51	183.82 ± 113.09	1122.11 ± 575.65
Diabetes	84	139.27 ± 27.74	187.53 ± 110.43	1185.46 ± 571.37
Nondiabetes	173	136.24 ± 28.40	180.16 ± 116.00	1117.47 ± 607.32
Smoking	101	139.34 ± 27.12	191.92 ± 115.31	1189.15 ± 573.52
Nonsmoking	156	135.79 ± 28.84	176.33 ± 113.31	1105.94 ± 609.84

Note: the data are given as the mean ± SD.

**Table 5:** Spearman's correlation analysis.

	IL-37	IL-18	IL-18BP
	(pg/mL)	(pg/mL)	(pg/mL)
TC (mmol/L)	0.02	0.08	0.11
TG (mmol/L)	0.00	0.01	0.03
LDL-C (mmol/L)	0.00	0.10	0.09
HDL-C (mmol/L)	−0.05	−0.15*	−0.10
GLU (mmol/L)	0.19	0.09	0.12
Creatinine (*μ*mol/L)	0.01	0.06	0.00
CRP (mg/L)	0.15*	0.24**	0.29**
NT-proBNP (pg/mL)	0.57**	0.63**	0.55**
LVEF (%)	−0.42**	−0.36**	−0.24**
LVEDD (mm)	0.34**	0.30**	0.20**
Gensini score	0.04	0.07	0.09

Note: *P < 0.05, **P < 0.01.

## Abbreviations

ACS: Acute coronary syndromes
ACEI: Angiotensin-converting enzyme inhibitor
AMI: Acute myocardial infarction
ARB: Angiotensin receptor blocker
CCB: Calcium channel blocker
CAD: Coronary artery disease
CRP: C-reactive protein
GLU: Fasting glucose
HDL-C: High-density lipoprotein cholesterol
IL: Interleukin
LDL-C: Low-density lipoprotein cholesterol
LVEDD: Left ventricular end-diastolic dimension
LVEF: Left ventricular ejection fraction
NT-proBNP: * N*-terminal pro-brain natriuretic peptide
PBMCs: Peripheral blood mononuclear cells
RT-PCR: Real-time quantitative reverse transcription-polymerase chain reaction
SAP: Stable angina
TC: Total cholesterol
TG: Total triglycerides
UAP: Unstable angina.
